# Laser-assisted drug delivery of topical ruxolitinib for treatment-refractory stable nonsegmental vitiligo: A case analyzed using noninvasive imaging modalities

**DOI:** 10.1016/j.jdcr.2025.11.039

**Published:** 2025-12-05

**Authors:** Bassem Rafiq, Gaurav N. Pathak, Noah Musolff, Madeline Tchack, Babar K. Rao

**Affiliations:** a Department of Dermatology, Rao Dermatology, Atlantic Highlands, New Jersey; b Department of Dermatology, Rutgers Robert Wood Johnson Medical School, Somerset, New Jersey; c Department of Dermatology, Weill Cornell Medicine, New York, New York

**Keywords:** combination therapy, Fraxel laser, LC-OC, reflectance confocal microscopy, ruxolitinib, skin pigmentation, vitiligo

## Clinical presentation

Vitiligo is a chronic immune-mediated disease characterized by the loss of pigmentary melanocytes in the epidermis, primarily mediated by cytotoxic CD8+ T-cells.^1^ A 24-year-old female patient with nonsegmental vitiligo of the face who had previously failed topical pimecrolimus and tacrolimus took part in our study investigating laser-assisted drug delivery (LADD) of topical ruxolitinib. The nonablative laser approach was utilized due to a lower risk of scarring, dyspigmentation, infection, and reduced downtime.^2^ She underwent 2 treatment sessions with fractional nonablative 1540 nm erbium glass laser, spaced 4 weeks apart, along with twice-daily 1.5% ruxolitinib cream. The eyelids were not treated; however, the patient wore protective eye goggles throughout the session for additional protection. Pretreatment imaging with reflectance confocal microscopy (RCM), line-field confocal optical coherence tomography (LC-OCT), and standardized VISIA photography was conducted during the initial visit before any intervention was initiated and at 4 and 8 weeks of treatment.

## Confocal microscopy appearance

**Fig 1:**
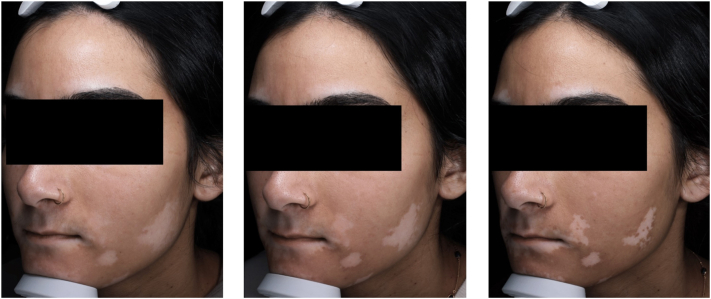
Clinical VISIA photos of vitiligo before treatment (*left*), after the first treatment at 4 weeks (*center*), and after the second treatment (*right*).

**Fig 2:**
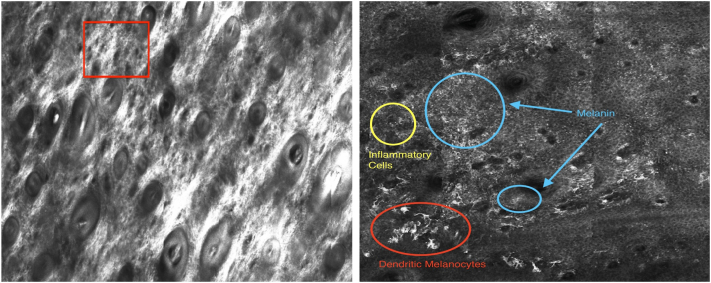
Reflectance confocal microscopy (RCM) imaging before and after laser-assisted ruxolitinib topical therapy. En-face RCM views of the affected vitiligo lesion along the left cheek or jawline before (*left*) and after (*right*) treatment. The RCM pretreatment image is at the level of the dermal-epidermal junction. The normally brightly marked papillae demonstrate absent pigmentation (*red square*). Posttreatment RCM imaging includes the presence of dendritic melanocytes (*red circle*), inflammatory cells (*yellow circle*), and melanin (*blue circle*).

**Fig 3:**
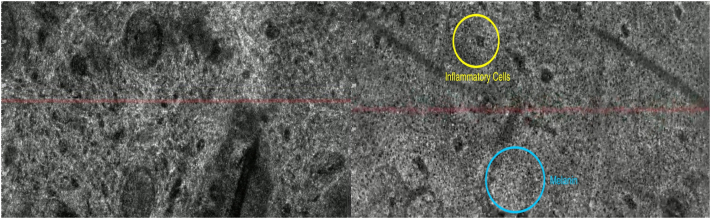
Line-field confocal optical coherence tomography (LC-OCT) images before and after laser-assisted ruxolitinib topical therapy. En-face LC-OCT views of the affected vitiligo lesion of the left cheek or jawline before (*left*) and after (*right*) treatment. Posttreatment imaging shows the presence of some inflammatory cells (*yellow circle*) and melanin (*blue circle*).

Three vitiligo lesions were treated with lasers and ruxolitinib, and the patch along the left jawline was monitored with RCM and LC-OCT (Fig 1). The patient experienced no adverse effects of topical treatment or following the laser administration. VISIA images show marked improvement in repigmentation of the treated areas, particularly after the second treatment. Pretreatment RCM findings included minimal melanin in the epidermis and dermo-epidermal junction (DEJ) with few inflammatory cells. Dendritic melanocytes were absent, and there were no capillary loops (Table I). LC-OCT imaging revealed similar findings except for the absence of melanin in the epidermis and DEJ. After treatment, RCM findings included marked melanin in the epidermis and DEJ, with inflammatory cells and moderate dendritic melanocytes (Fig 2, Table I). Posttreatment LC-OCT images had similar findings except for minimal dendritic melanocytes and moderate inflammatory cells (Fig 3, Table I).

**Table I:** Combined evaluation of vitiligo lesions using reflectance confocal microscopy and line-field optical coherence tomography

Parameter	Reflectance confocal microscopy		Line-field optical coherence tomography	
	Pretreatment	Posttreatment	Pretreatment	Posttreatment
Melanin: epidermis	Minimal	Marked	Absent	Marked
Melanin: dermo-epidermal junction	Minimal	Marked	Absent	Marked
Dendritic melanocytes	Absent	Moderate	Absent	Minimal
Inflammatory cells	Minimal	Marked	Minimal	Moderate

Scale of absent, minimal, moderate, and marked.

## Discussion

In this study, we reported the efficacy of LADD in enhancing the efficacy of ruxolitinib in nonsegmental vitiligo. Laser treatment can increase interleukin (IL) 4, IL-10, IL-17, and IL-23 levels, restoring the T-cell balance in the immune system, evidenced using inflammatory markers in our images.^1^ By altering the skin barrier up through the dermis, microscopic vertical canaliculi can enhance topical medication penetration at the active site in affected skin lesions. Similarly, the Janus kinase-signal transducer and activator of transcription signaling pathway that activates inflammatory cytokines and CD8+ T-cells is inhibited with ruxolitinib.

Noninvasive diagnostic imaging can be helpful for treatment monitoring and visualization of changes occurring at the cellular level in real time with objective and quantitative data.^3^ For example, although our patient did not see much clinical improvement at week 4, RCM pr LC-OCT imaging found some evidence of inflammatory cells and melanin, potentially avoiding premature treatment discontinuation.

Although LADD may also increase drug exposure and, theoretically, drug-related adverse effects, we found it was safe in this study.^4^ Limitations to this study include a limited sample size, lack of a comparator group of ruxolitinib alone, and short follow-up duration. RCM or LC-OCT limitations include high cost, low availability, limited field of view, and low penetration depths.^5^ Future studies can evaluate larger patient cohorts and other treatment modalities.

## Conflict of interest

None disclosed.
